# A New Argument for No-Fault Compensation in Health Care: The Introduction of Artificial Intelligence Systems

**DOI:** 10.1007/s10728-021-00430-4

**Published:** 2021-03-21

**Authors:** Søren Holm, Catherine Stanton, Benjamin Bartlett

**Affiliations:** Department of Law, School of Social Sciences, The University of Manchester, Manchester, U.K.

**Keywords:** Artificial intelligence, Deep learning, Clinical negligence, No-fault compensation, Tort, Product liability

## Abstract

Artificial intelligence (AI) systems advising healthcare professionals will be widely introduced into healthcare settings within the next 5–10 years. This paper considers how this will sit with tort/negligence based legal approaches to compensation for medical error. It argues that the introduction of AI systems will provide an additional argument pointing towards no-fault compensation as the better legal solution to compensation for medical error in modern health care systems. The paper falls into four parts. The first part rehearses the main arguments for and against no-fault compensation. The second explains why it is likely that AI systems will be widely introduced. The third part analyses why it is difficult to fit AI systems into fault-based compensation systems while the final part suggests how no-fault compensation could provide a possible solution to such challenges.

## Introduction

Discussions about whether the constituent parts of the United Kingdom, or other jurisdictions with tort/negligence based legal approaches to compensation for medical error^1^ should move to no-fault compensation systems have been ongoing for years. The UK and the USA have remained largely with tort/negligence systems,^2^ whereas New Zealand, Sweden, Denmark and others have introduced no-fault systems [58, 59].^3^ This paper is not aimed at resolving these discussions. It does not provide a definitive argument either way. It is more modest in scope and merely argues that the introduction of artificial intelligence (AI) systems in health care provides additional arguments pointing to no-fault compensation as the better legal solution to compensation for medical error in modern health care systems. It also provides a contribution to ongoing academic debates about liability for AI error in the health care setting.

The paper falls into four parts. In the first part we briefly rehearse the main arguments for and against no-fault compensation schemes. The second part explains why it is likely that AI systems advising health care professionals (HCPs) on issues such as diagnosis, prognosis, and treatment and care planning will be widely introduced into health care settings within the next 5–10 years. It describes the main features of current AI architectures and gives an account of the likely conditions for acceptance of AI systems in health care settings. The analysis is limited to advisory AI systems. Compensation for errors made by AI systems that make decisions, by AI controlled surgical or care robots, or by AI systems that are used directly by patients/clients/consumers are outside the scope of this paper. The third part provides an analysis of why it is difficult to fit AI systems into fault based compensation systems and an overview of the solutions to this problem. It argues that none of the solutions are satisfactory, and that no-fault systems can handle the issues created by AI systems in a much more straightforward way. Throughout the paper analogies are explored to liability and compensation issues created by the use of AI systems in other settings than health care. The analysis is not jurisdiction specific (though it draws on certain jurisdictions for examples), but relies primarily on the more general legal and philosophical principles underlying tort law.

## Fault and No-Fault Compensation for Medical Error

It is generally accepted by both sides of the debate about no-fault compensation schemes that tort/negligence based schemes are problematic because they require (1) adversarial litigation, (2) proof of breach of duty, and (3) proof of causation of foreseeable damage. Litigation takes time and resources and the adversarial nature of clinical negligence litigation is not conducive to an open discussion between patients and health care professionals about what went wrong and why [30, pp.74–5]. Establishing breach of duty can be difficult, as can showing causation, even in cases where something clearly went wrong but where it is difficult to pin the responsibility to one particular agent [24] or where causation is complex, e.g. because errors have been made by several agents employed by different organisations [25].

To apply this to the context of this paper: where a patient brings a claim about their care which involved AI, the practical challenges are significant. Firstly, who should they sue? In England this could include the hospital Trust as employer of the doctor who may be alleged to have negligently followed the advice of the AI system. Alternatively, this might be the Trust as directly liable for providing a safe system of care [7]. Should the claimant also sue the developer and/or manufacturer of the AI system? As we shall see, the nature of AI means that for the claimant it may be challenging to work out who is potentially at fault and therefore who should be sued. Investigation of all these issues may take time and will add to the already significant costs of instigating a personal injury claim.

No-fault compensation is designed to solve problems associated with negligence-based schemes by providing compensation for unexpected harmful outcomes of health care without the need for litigation or showing that any particular health care professional or part of the healthcare system was at fault. The process is designed to be non-adversarial and faster than litigation.^4^ The non-adversarial nature of the process, and the removal of the need to show fault is supposed to make it easier for HCPs and healthcare providers to report errors [21, p.14] and to take part in a reconciliation process which includes patients receiving explanations and apologies from HCPs and healthcare providers [46]. Supporters of no-fault compensation thus claim that it is better for all parties, patients, HCPs and the health care system.

But no-fault systems also raise problems. The problem that most often scuppers the introduction of no-fault systems is the projected cost of the system.^5^ If all patients with unexpected harmful outcomes of health care become eligible for compensation, costs are likely to go up, even if there are savings on litigation costs. There are also threshold setting problems in relation to both what makes an event unexpected (thus meaning that there is still a requirement to prove something similar to causation) and in relation to setting the limit for the lowest amount of harm that should receive compensation. It is also argued, that since there is no allocation of blame within no-fault systems, they reduce the deterrent effect of clinical negligence litigation [28, p.10].^6^

While it is not the aim of this paper to set out in detail the various no-fault schemes in operation, a brief examination of the New Zealand scheme serves as an example of how such a process can be set up, together with its advantages and challenges. The New Zealand scheme began in 1974^7^ and covers all personal injury, including medical injury. In 1992 reforms to the scheme meant that patients had to demonstrate ‘medical error’ in order to receive compensation. However, in 2005 this was reversed so that the scheme is now ‘no-fault’ [58, pp. 33–34]. Those injured cannot sue for damages, but receive payments from the scheme for unexpected treatment injury.^8^ This is defined as injury that is not a ‘necessary part or ordinary consequence, of the treatment’ taking into account all the circumstances, including the person’s ‘underlying health condition at the time of the treatment’ [11, s.1]. This is an easier standard to meet than traditional causation in negligence claims given that it does not require the claimant to demonstrate a causal link between the action and the harm, but just that the injury was not a usual consequence of treatment. Claims handlers, working for the Accident Compensation Corporation which administers the scheme, gather the necessary evidence [37, p.391]. After this evidence has been assessed, patients receive a written decision, which is then open to appeal [37, p.392]. As the scheme covers all personal injury, there are various accounts funded in different ways, which then pay out compensation. ‘Treatment injury’ is paid out of the earner’s account (funded by a levy on the employed) and the non-earner’s account (funded through general taxation) [12]. The reforms have increased access to compensation, but have also increased the cost of compensation for medical injury [58, p.41].

As we will argue, the introduction of AI provides a new argument for introducing no-fault compensation into health care. To understand why, it is important to first set out how AI is involved in health care provision.

## AI in Health Care

AI systems developed to provide advice to HCPs on issues such as diagnosis, diagnostic imaging, prognosis, treatment choice, and treatment or care planning have, according to their developers, ‘been on the cusp of a breakthrough’ ever since the first systems were designed in the 1950s. But, this time there seems to be some substance behind (some of) the hype. Although a lot of the research providing evidence for the performance of AI systems is of questionable quality, there is enough good quality research to allow us to predict with reasonable certainty that there will be areas of diagnostic imaging and of treatment planning where AI systems will outperform even experienced HCPs [43, 56]. The AI systems will have greater diagnostic accuracy or on average provide a treatment plan better suited to the patient’s condition than the HCPs who are currently making the diagnosis or proposing the treatment plan. Such systems may be directly embedded in a particular piece of diagnostic equipment or in an electronic patient record system, or they may be stand-alone. Simple AI systems are already embedded in many defibrillators and ECG equipment to interpret ECG traces and simple rule bound AI systems are embedded in EPR systems to generate warnings about potential drug interactions—GOFAI (Good old-fashioned AI). More than 30 AI systems, primarily analysing diagnostic images have already been approved by the US Food and Drug Administration, and similar systems are in use elsewhere in the world [57].

Advisory AI systems are likely to be introduced in health care systems when they have been shown convincingly to perform as well as or better than the average, experienced HCP in the relevant field. What we are likely to see is not the introduction of one overarching super AI being better than HCPs at diagnosing and treating patients across the whole of medicine, or even across some sub-specialty. The most likely development is the piecemeal introduction of AI systems that are very good within a particular well circumscribed area of diagnostics or treatment planning. To put this slightly differently, AI systems will be introduced when they make fewer errors than HCPs, not when they are perfect.

The potential to use AI is already apparent in various areas and it will probably be useful to provide a few examples. In 2016, Moorfields Eye Hospital NHS Foundation Trust entered into a research partnership with DeepMind (now owned by GoogleHealth)[9]. The project used an AI algorithm to detect and diagnose serious eye conditions from the 5000 optical coherence tomography scans (OCT) that are performed every week. The AI focussed on 53 key diagnoses relevant to NHS pathway referrals. The system was accurate 94% [8] of the time and the performance in making recommendations ‘reaches or exceeds that of experts on a range of sight-threatening retinal diseases after training on only 14,884 scans’ [29].

Image recognition is currently the most mature of AI diagnostic technologies and has also shown potential in interpretation of head CT scans [18], as well as in the diagnosis of malignant tumours in breasts [35], lungs [32], skin [16] and brain [34]. Deep convolutional neural network-based software (DCNN) can be used to classify images and help clinicians interpret scans. Decision-making with support from DCNN-software has been shown to improve the performance of even experienced radiologists [52].

AI has also shown credible potential to impact patient care in planning treatment. One possible avenue of deployment is in the treatment of sepsis, which is the third leading cause of death worldwide, as well as the most common cause of mortality in hospitals. Sepsis treatment requires careful management of intravenous fluids and vasopressors and suboptimal decision-making leads to poorer outcomes. Komorowski et al. [39] used a reinforcement learning agent to examine a large dataset and the results showed that the treatment selected by the AI clinician was on average reliably better than human clinicians. There is much hope that computational models like this can enhance clinical decision-making and improve patient outcomes in the future.

The latest published horizon scanning exercise carried out by the NIHR Innovation Observatory for the NHSX report shows that there are now 132 AI products that have been developed, covering 70 different conditions [47].

The AI systems we are discussing here are advisory. They do not make decisions about diagnoses or treatment plans. They advise HCPs who then make decisions. In the future we may introduce AI systems in health care that make decisions without the involvement of HCPs in the decision-making loop. In the EU, the introduction of AI decision-makers would be in conflict with the right "not to be subject to a decision based solely on automated processing” that is guaranteed by Article 22 of the General Data Protection Regulation (GDPR) if the decision significantly affects a person [14]. This prohibition does, according to the official Data Protection Working Party apply to “decisions that affect someone’s access to health services”, and the human involvement has to be meaningful and “it should be carried out by someone who has the authority and competence to change the decision. As part of the analysis, they should consider all the relevant data” [15, pp.21 & 22].

It is, however important to consider whether our bland statement above that ‘they advise HCPs who then make decisions’ is likely to be an accurate description of how AI systems will actually function in health care settings. There is a significant literature on automation bias (AB) in relation to decision support systems, i.e. the cognitive bias:

… by which users tend to over-accept computer output ‘as a heuristic replacement of vigilant information seeking and processing.’ AB manifests in errors of commission (following incorrect advice) and omission (failing to act because of not being prompted to do so) when using CDSS [Clinical Decision Support Systems][31, p. 121, reference removed].

Automation bias has been shown to be quite prevalent in medical decision-making, both in experimental settings and in actual practice. It can be mitigated in various ways, but cannot be reliably removed. High workload, task complexity, and time constraint on the decision-making process makes AB more likely to occur, as does trust and confidence in the system providing the advice.

So, let’s imagine a junior doctor in a busy accident and emergency department evaluating a newly arrived patient with chest pain. After having gathered all the relevant data (history, physical examination, ECG, blood tests etc.) she follows the department’s guideline and uses the newly acquired ‘AI system for chest pain diagnosis and treatment advice’. She has been informed and believes that the system has a diagnostic accuracy better than the average cardiology specialist. The system tells her that there is a 76 percent likelihood that the patient has condition A and should therefore be treated with treatment Ta, a 13% likelihood of B with best treatment Tb, and a combined 11% likelihood of a range of less likely conditions C-J with treatments Tc-j. A is not an unlikely diagnosis and the patient’s history and symptoms are broadly compatible with A. After having received this advice she decides that the patient’s diagnosis is A and that treatment Ta should be commenced. Unfortunately, it turns out that the AI system gave the wrong advice, i.e. made a medical error, and that the patient really has condition E and that Ta is very risky and therefore contraindicated for patients with E. In this hypothetical scenario it is true in one sense that the decision about diagnosis and treatment (the medical error) is made by the junior doctor, and that she, according to the standard principles of negligence is negligent if the right decision would have been made by an A&E specialist. But, in another sense she was set up for making the medical error by the conditions under which she has to work which make decision-making time-constrained, and in addition by being explicitly directed to use and have confidence in the AI system. To the junior doctor, overruling the advice of the AI system may phenomenologically be experienced as similar to overruling advice given over the phone by a senior colleague.

We are not suggesting that HCPs should not think carefully about whether the advice they are being given by an AI system is valid and sound advice. It is perfectly possible that a HCP could be negligent by blithely accepting and acting on AI advice that is very obviously flawed. What we are suggesting is that there are many circumstances where it is predictable that even a conscientious HCP may end up acting on erroneous advice.

There are many other contexts in health care where AI systems will be used by HCPs who are not at the top of the HCP hierarchy and where the HCPs will (rightly) believe that the AI system providing them with advice on average performs better than they do at the particular classification task at hand. In such contexts it would be perverse not to accept that HCPs are likely to over-rely on the AI advice, and that they are furthermore not negligent in most cases where they do over-rely. Yet, as we explain in the next section, there are reasons why the AI advice may contain errors.

## AI Explainability and Error

### Errors Relating to Data

AI systems may make errors for a number of different reasons. The data about the patient that is available to the system may contain errors, be of poor quality, or be incomplete and this may, according to the (in)famous GIGO principle^9^ lead to erroneous decisions. Such errors are only errors of the AI system if the system ought to have detected the error or incompleteness in the input data.

For example, an algorithm was designed to predict the probability of death amongst hospital patients with pneumonia [17]. Patients with asthma were systematically classified as low-risk by the algorithm. This calculation was fundamentally flawed because asthma patients are routinely sent to the ICU where the continuous intensive treatment improves their prognosis, thereby making it appear that they have a better chance of survival. The different clinical pathway skewed the output of the computational model and shows the potential for similar errors—many recurrent error traps may be laid within spurious correlations. This example appears to be owing to the ‘brittle’ [44] nature of AI; that ‘they can’t be trusted with anything that hasn’t been precisely anticipated by their programmers. That is particularly important when the stakes are high’ [44, p.15].

In addition, bias in training data is highlighted as a risk because the data sources themselves may not reflect the true epidemiology within a given demographic [49]. A significant challenge to safety exists because an error rate may be far more likely to occur in under-represented groups such as ethnic minorities [42] those with disabilities [60] and women [20]. Machine learning (ML) classifiers improve with volume of data and proportionately less data exists for minorities [33]. In the US an algorithm used to allocate healthcare resources had been widely discriminating against African Americans; subsequently, they were far less likely to be referred for treatment than white people when equally sick [48]. Another example is that skin cancer detection algorithms may be less effective on darker skin [13, 40].^10^ If ML learning algorithms are not transparent, then it could be difficult to establish where a specific error occurs within the AI lifecycle. If inaccuracies are not picked up in a learning AI system, a feedback loop could be created which may exacerbate existing inequalities and create self-fulfilling prophecies [51].

## Errors Due to the System

Other errors are not due to problems with the input data, but are caused by the internal workings of the system. The data are correct and complete, but the system reaches the wrong decision. It is primarily this type of error that is of interest here because it can be unequivocally ascribed to the AI system itself.

Traditional AI systems^11^ attempted to emulate an idealised model of clinical decision-making and were often built around an architecture based on Boolean logic with branching, explicit rules, e.g. ‘IF the patient has a temperature >  = 38.5 °C AND the patient coughs up green sputum THEN order chest X-Ray’. Such systems are fully transparent and their decision making fully explainable because their rules are explicit and refer to the kind of symptoms and signs that are part of ordinary and medical language. It did, however over time become evident that it was difficult to build AI systems with this kind of architecture that could compete in performance against HCPs.

The breakthrough in AI in medicine and in general, came with the development in the late 2000s of new data-driven, self-learning architectures such as neural networks and Support Vector Machines; and the rapid increases in computational speed and the availability of large data sets that made it possible to train and run such architectures efficiently.

These self-learning architectures are, for instance the basis for speech recognition systems like Google’s Assistant, Amazon’s Alexa or Apple’s Siri. A self-learning architecture ‘learns’ how to perform a particular classification task from a large already annotated/classified data set, e.g. pictures of moles classified into malignant and non-malignant. By then running the developed classifying AI system/model on another large annotated data set, which the system has never seen before, it becomes possible to estimate its performance in terms of the accuracy of classification.

The new self-learning architectures are in principle transparent. For any given decision to classify a case in a particular way it is possible to go through the precise mathematical calculations that link the input data to the output, i.e. the classification. But despite being technically transparent the decisions are not interpretable or explainable because (1) the mathematical model is highly complex, and (2) the mathematical model gives weight to features in the data that are unexplainable in natural language. Mittlestandt et al. note that even the designers of the technology cannot provide a human comprehensible representation of them [45]. Deep learning makes predictions, but not explanations. This intrinsic opacity of machine learning systems explains why they are generally referred to as ‘black boxes’. It is also important to note that some AI systems use proprietary algorithms which mean that access to explanation when an error has happened may be hindered by commercial secrecy considerations [45, p.6] [38].

Issues surrounding AI explainability and error outlined above raise challenges if a tort-based system is used to compensate harm. Whereas it may be possible to establish liability against the manufacturer in tort where it is apparent that a failure has occurred due to the inadequacy of the training set, an internal error presents a different scenario. How should we decide whether the classification error should lead to legal liability for compensation in those cases where it leads to harm to a patient, if we cannot explain why or how the error happened? A patient seeking to bring a claim in tort against a manufacturer would have to show they were owed a duty of care, that this was breached and that the damage was foreseeable [1]. Yet, as Dodd has argued:

With the human players (programmers, manufacturers, sellers, etc.) totally removed from the decision-making process of AI solutions, the argument that a harmful outcome is foreseeable falls short [23].^12^

## Possible Solutions within a Fault-Based System

As we have seen, there are problems with using a fault-based system to compensate harm caused to patients in the context of AI. These include: (1) establishing who may have been at fault and therefore who to sue ie the HCP, the software designer or the manufacturer of the system, (2) establishing whether any error caused by the AI system was due to data error or a systems error, (3) where the problem is a system error, demonstrating foreseeability. Within a fault based system there are some possible solutions to the liability issues raised by advisory AI systems [54]. But, as we will show they are all problematic in various ways.

The first is to maintain the legal fiction that AI systems are only ever advisory and that the legal liability therefore always falls to the HCP who negligently did not realise that the advice given was wrong and would lead to harm to the patient. As we have argued above it is very unlikely that HCPs will be able to pick up all or even most of the errors made by an AI system in daily clinical practice. This is especially the case when that AI system is believed by the HCPs to be performing better than they do, and/or when they been instructed to use it on the basis that ‘it performs better than you do’. If that turns out to be true of experienced HCPs at the top level of the professional hierarchy it will become very difficult for a patient to prove negligence if an AI error is not identified, unless there is something specific about the error that makes it obvious that it is an error. This means that although the frequency of errors leading to harm should *ex hypothesi* fall if doctors follow the advice of highly accurate AI systems, the patient’s possibility of getting compensation when an error has demonstrably occurred will diminish.

A second option is to draw on existing legal principles to assist claimants. It has been suggested that the doctrine of *res ipsa loquitur* (the thing speaks for itself) could be helpful in this context [51, p,197]. This doctrine, applied in various jurisdictions including the UK, has been applied in medical negligence claims where it is difficult to establish negligence. The doctrine creates an inference of negligence and if the defendant cannot rebut this, then the claimant will succeed [41, pp. 141–3]. However, more recent case law has suggested that *res ipsa loquitur* is not in fact a principle of law, but that so called *res ipsa loquitur* cases are just those where the court was able to find negligence based on inference from the available evidence [2]. It does not appear that this principle (if it exists) would necessarily assist claimants to resolve the challenges of litigation in this context. If a patient sued a hospital Trust alleging that the AI system used in their care was to blame for the harm caused, then the Trust would no doubt (in the absence of obvious breach of duty on the part of the HCP) join the manufacturer/developer of the AI system into the proceedings. The claimant would then become embroiled in complex litigation in a bid to seek an explanation for the harm caused. The claimant would have to determine whether the harm caused was due to an error relating to data used or the internal workings of the system. As we have seen above, if it were the latter, there might be challenges in demonstrating foreseeability. Therefore, while the evidence (or lack of it) gathered might assist the court to make inferences at trial, the principle does not address the challenges for the claimant in undertaking the litigation process (ie the cost and time involved), nor assist where the harm arose due to a systems issue which is deemed unforeseeable.

The third option is to move to the kind of strict liability found in some areas of consumer law. If the errors made by a particular system are systematic in the sense that the system is more likely to misclassify some identifiable type of patients, either because of bias or inadequacy in the training set, or because of a design flaw in the implementation of the general self-learning algorithm, then strict product liability could be a more promising approach to obtaining a legal remedy for any harm caused by misclassification than tort. In product liability there is no need to show the existence of a duty of care or that harm was foreseeable, there is only a need to show that harm was caused by a defective product. An AI system that generates systematic errors is arguably defective and the developer or producer arguably responsible for this defect. In the UK such a claim would be brought under the Consumer Protection Act 1987 (CPA) and subsequent amendments. Product liability regimes differ considerably between jurisdictions and providing a detailed analysis of their application to advisory AI in health care is beyond the scope of this paper [27, 50]. It is, however important to note some of the general problems a claim might meet if made in product liability. The first issue is that ‘pure’ software may not be deemed to be ‘a good’ in some jurisdictions where it is therefore excluded from the scope of product liability legislation [3]. This question is still contested in the literature [36]. The second and more widely relevant issue is that product liability regimes often primarily protect the consumer who suffers harm from a defective product, but the patient is very rarely the direct consumer in relation to the advisory AI system. The hospital or the clinic is the consumer. Even if we extend the class of consumers to include the users of the system, this does still not encompass the patient who has suffered harm. Some jurisdictions also take account of the involvement of a learned intermediary such as a health care professional in the chain of causation between product and harm when deciding whether the product caused the harm [4]. The third issue concerns the concept of ‘defect’. The CPA definition of defect is seemingly very simple and straightforward: “…there is a defect in a product […] if the safety of the product is not such as persons generally are entitled to expect…”[19, s. 3(1)]. However, the Act provides a development defence that will be relevant to advisory AI in the foreseeable future: “that the state of scientific and technical knowledge at the relevant time was not such that a producer of products of the same description as the product in question might be expected to have discovered the defect if it had existed in his products while they were under his control;…” [19, s. 4(1)(e)].^13^ Until legal standards for responsible AI development have been established specifying what is expected in law of a developer and producer of AI it will not be clear when a producer “might be expected to have discovered the defect”. Even if all of these issues can be resolved strict product liability is not likely to be a successful avenue for compensation claims if the errors made by the AI system are not systematic and not more frequent than for other similar AI systems developed at the same time, since the mere fact that an AI system made errors would not make it defective.

The fourth potential solution is to give AI systems legal personality making it possible to sue the system itself for negligence.^14^,^15^ This in turn would require either AI developers or AI owners to insure their AIs against this potential liability,^16^ since the AI system would be penniless and could not itself pay out compensation if successfully sued. Perhaps more importantly this approach gives rise to very complicated and philosophically intricate questions about what ‘negligence’ or ‘failing in a duty of care’ means in relation to a mathematical model instantiated as a piece of software running on a computer. A doctor is not negligent or failing in a duty of care simply because he or she makes a wrong decision that leads to harm to the patient. The decision has to be one which a responsible doctor in the same position would not have made. As McNair J. set out in the landmark case of *Bolam*, a doctor will not be liable for negligence where he or she acted:

[…] in accordance with a practice accepted as proper by a responsible body of medical men skilled in that particular art….a man is not negligent, if he is acting in accordance with such a practice, merely because there is a body of opinion who would take a contrary view [5, p.587].

This test has now been given a gloss following the case of *Bolitho*, so that the court, rather than the profession is the final arbiter of responsible practice. As stated in that case: ‘the court has to be satisfied that the exponents of the body of opinion relied upon can demonstrate that such opinion had a logical basis’[6, p.241–2].

But what is the relevant comparator for an AI system? Another similarly situated AI system or a medical doctor? The relevant comparator cannot be a medical doctor. We introduce AI systems in the full knowledge that they use a different logic than humans do in order to reach decisions. We also know that use of mentalistic language like ‘should have known’ or ‘must have realised’ is inapplicable in relation to AI systems or at best highly metaphorical. AI systems are not conscious and do not have minds. But, if AI systems are mindless and if they work according to a fundamentally different logic than humans when they perform their classification tasks, it makes no sense to compare with a human decision-maker and say that the error was obvious, i.e. obvious to a human HCP, because it is highly unlikely to be ‘obvious’ within the logic used by the AI system. The AI system is not negligent, i.e. malfunctioning in any way. It is not inattentive or applying a decision-making model it should not apply. We could then say that the relevant comparator is another AI system. If another AI system would not have made this error then the AI system which is being sued is negligent and liable in tort. There are two problems with this suggestion, one practical and one conceptual. The practical problem is that the number of providers of AI systems within a particular clinical domain may be quite limited due to the need to access large datasets for the development of the systems and due to commercial constraints, so there may not be any other AI system with which to compare. The sheer intensity of computing resources and the requirement for access to very large data sets currently required for further breakthroughs may leave the monopolistic tech giants as sole custodians of the tech. There is some evidence that resource constraints is already privatising research in AI [53] and even pricing academia out of research.

The conceptual problem can be illustrated by considering a hypothetical situation where we have two distinct AI systems^17^ that have similar accuracies, e.g. both correctly identify 97% of those patients who have a particularly nasty type of cancer and who need a very specific treatment. From the fact that both only make 3 errors out of each 100 diagnoses does not follow that they make identical errors, i.e. that they misclassify the same three patients. Because distinct AI systems will instantiate different mathematical models it is likely that they will misclassify different patients. They will classify the core patient who has all the paradigmatic features of the condition in the same way, but away from the core they may classify differently. This means that the fact that two equally effective AI systems disagree about a classification does not show that one of them has made an error, which a well-functioning AI system should not make. This hypothetical example also shows why a suggestion put forward in the US literature that we should apply a “reasonable computer” standard to determining AI negligence claims does not work [10]. Both the two AI systems are perfectly reasonable as AI decision-makers. It may be that the advice provided in some of the 3% of cases where the systems do not classify correctly would be an obvious error to a human expert, but that does not make the AI system a less reasonable computer. There may of course be situations where a health care institution uses a sub-optimal or obsolete AI system to provide advice and where there is a higher performing, more accurate and affordable system on the market. In that case there is a case for saying that a culpable error has been made if the AI system used does not make a diagnosis which the currently best available AI would have made. But, that error is importantly not an error attributable to the AI system, but an error attributable to the organisation that has not updated its technology.

As we have argued above none of the options for handling medical errors made by AI systems within a traditional tort/negligence system for compensation are appealing. Can a no-fault system do better?

## No- Fault System?

In a no-fault system the right to compensation depends on a positive answer to two questions, i.e. (1) was the health care outcome unexpected, and (2) was the unexpected outcome harmful? If an advisory AI system makes an error and the advice is followed and leads to harm then both of the criteria for compensation under a no-fault scheme have been met. The outcome is unexpected^18^ and harmful. This means that the patient has a right to compensation. If we adopted a no-fault scheme for all claims involving AI error (including those due to deficient training data which could, as argued above be pursued under existing law), we would not need to consider (1) exactly why the AI malfunctioned, e.g. was it a problem with the input or the internal model, or (2) why the HCP followed the advice, or (3) whether other HCPs similarly situated would have followed the advice, or (4) whether another AI system would have done better, because we do not have to locate any specific locus of responsibility and/or negligence before we can conclude that the patient should be compensated. This is much simpler than relying on proving negligence, and it removes the potential unfairness facing a claimant having to pierce the layers of technical (and potentially commercial) lack of transparency inherent in the decision-making algorithms of current AI architectures.

On a practical level this would make the task of gaining compensation much more straightforward and quicker for claimants. Imagine a patient who suffers harm while in hospital. When she makes a complaint to the healthcare provider she is advised that this was due to the AI system. Under a fault system, she (or her advisers) would then face having to investigate the issues set out above and determine who to sue ie was it the fault of the HCP in following the advice or was it due to the AI and if so, why? The latter questions as we have seen could be very complex to resolve. In contrast, under a no-fault scheme she would not need to decide who to sue and would instead be able to claim from a specific fund provided she could demonstrate injury which was an unexpected outcome of treatment.

This paper does not aim to set out how such a fund should function, but a scheme could be set up with contributions from the State and/or relevant stakeholders such as the designers and manufacturers of AI systems. The fund could also be assigned patients’ rights to take on recovery actions to recoup pay-outs where it was considered that the harm was caused by failures in the design and/or manufacturing of the AI systems. This would take the investigatory burden from the patient and also incentivise developers/manufacturers to address errors within their systems. There would be scope too for a hybrid system in relation to patients’ rights. So, for example if claims handlers deemed that the actions of a HCP in following the AI advice were a clear breach of duty, then the claimant might be advised to bring a negligence claim. However, such details would have to be determined when devising the scheme as a whole.

If the prediction is right that AI advisory systems are likely to permeate all aspects of health care delivery [56] an introduction of no-fault compensation for AI error will come to cover an increasingly larger fraction of all claims made, and in time it may pave the way for a complete switch to a comprehensive no-fault system.

## Footnotes

1 We use the term ‘medical error’ for any error made by a health care professional that leads to harm to a patient or a client.

2 Note the existence of ‘no fault’ schemes for birth-related neurological injury in Virginia and Florida: see Farrell et al., [28], pp.52–63.

3 Whereas New Zealand has moved to a ‘no-fault’ scheme whereby a patient cannot sue for damage covered by the scheme, other jurisdictions, such as Sweden retain the option to go to court. For a summary see Dickson [22, p. 9].

4 For a summary of arguments in favour of no-fault systems see [28], pp. 9–10.

5 This was one of the reasons for the rejection of a comprehensive no-fault NHS scheme following a report by the Chief Medical Officer in 2003. Another reason was concern that the scheme would have to comply with Article 6 European Convention on Human Rights which provides the right to a fair hearing. See [21, pp.111–113].

6 Though doubts have been raised as to whether a fault-based liability system acts as a deterrent. As Lord Sumption has commented: ‘The whole notion of deterrence assumes that there is a minimum of reflection behind the actor’s decisions. Negligence normally consists in the absence of the very processes of reflection which the notion of deterrence assumes. It generally happens through ignorance, incompetence or oversight, none of them states of mind which are normally associated with reflection upon the possible consequences’ [55, p. 118].

7 Accident Compensation Act 1972. There has been subsequent legislation including the Accident Compensation Act 2001.

8 Note that other ‘no-fault’ schemes do allow claimants the option to sue for damages or to claim compensation under the scheme. See [22].

9 Garbage In, Garbage Out.

10 Discussion of non-discrimination law is excluded from this paper but has been discussed elsewhere: see [51].

11 Often called’Expert’ or ‘Decision support’ systems in the literature of the 1960s-90 s.

12 Dodd is discussing foreseeability in the context of establishing a ‘duty of care’, but the same point can be made in relation to establishing the foreseeability of damage.

13 The European Commission has examined the issue of AI and product liability as part of its digital agenda [26].

14 Given the limited scope of the AI systems we are discussing here we are not engaging with the discussion of whether some future AI systems may be more real persons and individual legal right holders, but see e.g. Zenor, [61].

15 As highlighted by Dodd [23] this could also introduce the possibility of vicarious liability – ie where one person (eg the manufacturer of the AI system) becomes liable for the tortious act of another party (eg the AI system).

16 This presumes that commercial insurance would be available.

17 Distinct in the sense that they have been developed by different firms, have different architectures, and have been trained using different data sets.

18 That is, unexpected in the individual case, although as pointed out above expected since we know when introducing them that AI systems will make errors.
